# Use of presumptive recommendations and other strategies to encourage HPV vaccine uptake: Results from a national survey of primary care health professionals

**DOI:** 10.1371/journal.pone.0327872

**Published:** 2025-08-04

**Authors:** Anna A. Ilyasova, Tara L. Queen, Melissa Gilkey, Benjamin N. Fogel, Olufeyisayo O. Odebunmi, Juan Yanguela, Assanatou Bamogo, Yeshaben Patel, Erin Laurie, Sachiko Ozawa, Stephanie B. Wheeler, Lisa P. Spees

**Affiliations:** 1 Department of Medicine, University of North Carolina, Chapel Hill, United States of America; 2 Lineberger Comprehensive Cancer Center (LCCC), University of North Carolina, Chapel Hill, United States of America; 3 Department of Health Behavior, Gillings School of Global Public Health, University of North Carolina, Chapel Hill, United States of America; 4 Department of Pediatrics, Penn State College of Medicine, Hershey, United States of America; 5 Department of Health Policy and Management, Gillings School of Global Public Health, University of North Carolina, Chapel Hill, United States of America; 6 Department of Maternal and Child Health, Gillings School of Global Public Health, University of North Carolina, Chapel Hill, United States of America; 7 Division of Pharmaceutical Outcomes and Policy, Eshelman School of Pharmacy, University of North Carolina, Chapel Hill, United States of America; 8 Division of Practice Advancement and Clinical Education, Eshelman School of Pharmacy, University of North Carolina, Chapel Hill, United States of America; Yarmouk University, JORDAN

## Abstract

**Background:**

Primary care health professionals’ (PCHPs’) use of presumptive recommendations, which assume parents want to vaccinate, is associated with greater HPV vaccine uptake. We analyzed PCHP characteristics associated with using this and other strategies to encourage HPV vaccination to inform future communication interventions.

**Methods:**

A national sample of 2,527 PCHPs (26% pediatricians, 22% family physicians, 24% advanced practitioners, 28% nursing staff) completed our survey in 2022. PCHPs reported which of six communication strategies, including presumptive recommendation, they used to encourage HPV vaccination. Multivariable regression models identified PCHP characteristics associated with use of each strategy.

**Results:**

Overall, 58% of PCHPs used presumptive recommendations. Use of presumptive recommendations was more common among pediatricians (74%) than family physicians (57%), advanced practice providers (54%), or nursing staff (48%, all *p* < .05). Pediatricians were also more likely than nurses to use prepared talking points, patient stories, motivational interviewing, and offer vaccination another day to hesitant caregivers. PCHPs who had received training on how to introduce HPV vaccination and address parental hesitancy were more likely to use presumptive recommendations (65% vs. 55%, and 67% vs. 53%, respectively).

**Conclusions:**

Our findings suggest that PCHPs, particularly non-pediatricians, could benefit from additional training on evidence-based HPV vaccination communication strategies.

## Introduction

Human papillomavirus (HPV) vaccination is a highly effective way to prevent anogenital warts, oropharyngeal cancers, and over 90% of all anogenital malignancies, including vaginal, cervical, vulvar, and penile cancers [1,2]. National recommendations in the US support the two-dose HPV vaccine series at ages 9–12 [3]. Despite the safety and efficacy of HPV vaccination [4,5], only 61% of adolescents ages 13–17 were up to date in 2023 [6].

Effective recommendation from primary care health professionals (PCHPs) is positively correlated with HPV vaccine series initiation [7,8]. Certain communication strategies employed by PCHPs have been shown to be particularly effective at increasing HPV vaccine acceptance. For example, the presumptive recommendation encourages PCHPs to lead the conversation regarding HPV vaccination directly (“Today your son is due for the HPV vaccine.”) in contrast to more ambiguous approaches (“Would you like to get the vaccine today?”) [9–11]. Multiple randomized controlled trials have found that training PCHPs to use presumptive recommendations increases HPV vaccine uptake [12–16]. Once parents express hesitancy about HPV vaccine, PCHPs may use other communication techniques to encourage uptake, including use of motivational interviewing techniques (with personalized, open-ended questions) [12], fact sheets (such as family education materials) [13], and narrative communication [14] (story sharing). Primary care practices trained to use multi-component interventions, including the presumptive recommendation, motivational interviewing, fact sheets, and other communication strategies had an estimated 9.5% increase in HPV vaccine initiation compared to practices who did not utilize this approach [13].

A recent systematic review examined PCHP characteristics associated with consistent and high-quality HPV vaccine recommendations and found that pediatricians were consistently more likely to employ high-quality HPV vaccine communication with parents than physicians in family medicine [15]. However, few studies have examined PCHP characteristics associated with use of the presumptive recommendation specifically, one of the most effective and efficient approaches for increasing HPV vaccine uptake. Similarly, studies have yet to explore potential patterns in the PCHP characteristics associated across the various strategies used in counseling vaccine hesitant parents. The first objective of this study was to identify associations between PCHP characteristics and use of the presumptive recommendation and other strategies (such as motivational interviewing, providing educational materials) for HPV vaccine uptake among hesitant patients. Second, we sought to explore PCHPs’ previous communication training experiences, in relation to their current use of the presumptive recommendation.

## Methods

### Participants

Online surveys were administered from May 2022 through July 2022 to PCHPs who worked in clinics that provided HPV vaccination to children. Survey participants were those who 1) were currently licensed and practicing physicians, physician assistants (PAs), advance practice nurses (APN), registered nurses (RNs), licensed practical/vocational nurses (LPN, LVN), medical assistants (MAs), or certified nursing assistants (CNAs) in the United States: 2) specialized in pediatrics or family medicine, or worked in a clinic that focused in those areas; and 3) had a patient-facing role in the administration of HPV vaccination for children ages 9–12 years. Patient-facing HPV roles included assessing a child’s prior vaccination status, announcing that a child was due for HPV vaccination, discussing the benefits/risks of the vaccine, addressing questions and concerns, or directly administering the vaccine.

### Procedures

The survey was administered through WebMD Market Research, which generated a national sample of US PCHPs included in the Medscape Network directory [16]. Medscape Network members, which includes about 60% of US physicians, received an initial email describing the study with an invitation to participate. Those who indicated interest were asked to complete an eligibility screener. Eligible individuals were sent four reminders to encourage survey completion. Participants who completed the survey received a $30–45 honorarium, based on level of medical training. Among the 6,278 initial email recipients, 2,242 did not express interest or complete the eligibility screener, and 1,509 respondents were screened to be ineligible or did not complete the survey. The final sample included 2,527 PCHPs, with a response rate of 57% (AAPOR Response Rate 3). More detailed information on the survey recruitment and administration process is available elsewhere [16]. The University of North Carolina Institutional Review Board approved this study.

### Measures

Survey questions were modified after feedback from 39 cognitive interviews. In these pre-survey cognitive interviews, PCHPs were asked to explain their thoughts about and responses to the questions asked, and were encouraged to recommend changes to survey questions. For example, after asking cognitive interview participants about their use of the presumptive recommendation, we asked “Is there something that is unclear or confusing? Is there another way you would suggest asking this question?”. Responses were compared to the intended survey goals. Survey instructions and questions were updated for clarity and readability prior to administration.

#### HPV vaccine-related measures.

The survey assessed nine HPV vaccine-related outcomes. First, we created a binary variable indicating if participants reported that the presumptive recommendation was their most often used HPV vaccine communication strategy when bringing up HPV vaccine with parents of children ages 9–12. Specifically, responses options included: 1) I ask a question, such as: “How do you feel about HPV vaccine for your child today?”, 2) I assume routine vaccine acceptance and might say: “We’ll give vaccines today that protect against meningitis, HPV cancers, and whooping cough...”, 3) I bring up HPV vaccine only if asked about it. Use of the presumptive recommendation was represented by the second response option. Next, participants indicated which strategies they used with parents who were hesitant to get the HPV vaccine for their children ages 9–12 following use of the presumptive recommendation. Response options included: offering the option to get the vaccine another day, using prepared talking points, providing educational materials, sharing patient stories, and using motivational interviewing. Finally, we asked participants about whether they had had previous training on 1) how to bring up HPV vaccination, 2) how to address parental hesitancy, and 3) previous opportunities to practice through role play, specifically related to HPV vaccination.

#### PCHP and clinic characteristics.

PCHP characteristics collected included their gender, race and ethnicity, medical training, years of practice (inclusive of years in post-license specialty training, such as residency), as well as clinic-level characteristics, such as the number of patients age 9–12 seen in an average week. Self-reported race and ethnicity were categorized as Asian, Black, Hispanic/Latin/Spanish, White, multiracial (which included individuals who identified with two or more race and ethnic groups), and other/prefer not to say. Medical training was categorized as pediatricians, family medicine physicians, advanced practice providers (APP), or nursing staff. APPs included physician assistants, nurse practitioners, advance practice nurses, and clinical nurse specialists. Nursing staff included RNs, LPNs, LVNs, CNAs, and MAs.

We also collected information on the PCHP’s primary clinic, including whether the clinic was located in a rural area, designated as a federally-qualified health center (FQHC), or part of a larger healthcare network. We also reported on the number of providers in the clinic and the clinic’s geographical location (South, West, Midwest, Northeast). Clinic rurality was categorized based on US Department of Agriculture (ISDA) Rural-Urban Continuum Codes (RUCC) 4–9.

### Statistical analyses

We initially conducted bivariate logistic regressions to examine covariates associated with use of the presumptive recommendation as well as strategies used with hesitant parents. We then included covariates that were statistically significant at the bivariate level in our multivariate models. Average marginal effects and 95% confidence intervals were computed for each covariate (See S1 Table in S1 File for proportions).

Lastly, we used two-tailed chi-square tests with alpha of 0.05 to examine differences in prior training received between providers who used the presumptive recommendation and those who did not use the presumptive recommendation. Analyses were conducted in STATA version 13 (StataCorp, College Station, Texas).

## Results

### Sample characteristics

Our sample was comprised of similar proportions of pediatricians (26%), family medicine physicians (22%), advanced practice providers (24%), and nursing staff (28%) (Table 1). The majority were women (72%). Participants self-identified as Asian (14%), Black (5%), Hispanic (4%), White (66%), or multiracial (4%). Others described another ethnicity, or preferred not to say (8%). Over one-quarter (29%) of participants saw less than 10 patients ages 9–12 in a typical week, whereas 40% saw between 10–24 patients, and 32% saw greater than or equal to 25 patients. There was also variation in years of experience, with approximately one-third of participants reporting less than nine years (38%), 10–19 years (29%), and over 20 years (33%) of clinical practice (Table 1).

**Table 1:** PCHP and clinic-level sample characteristics (N = 2527).

	n	(%)
**PCHP Characteristics**		
Gender		
Man	637	25.2
Woman	1810	71.6
Another gender^a^	80	3.2
Race/Ethnicity		
Asian	356	14.1
Black	123	4.9
Hispanic/Latine/Spanish	100	4.0
White	1664	65.8
Multiracial^b^	94	3.7
Other/preferred not to say	190	7.5
Medical training		
Physicians		
Pediatrican	666	26.4
Family medicine	557	22.0
Advanced practice providers^c^	603	23.9
Nursing staff^d^	701	27.7
Patients ages 9–12 seen in a typical week		
< 10	730	28.9
10–24	1000	39.6
≥ 25	797	31.5
Years of practice		
0–9	950	37.6
10–19	740	29.3
≥ 20	837	33.1
**Clinic Characteristics**		
Clinic Rurality		
No	2295	90.8
Yes	232	9.2
FQHC or health department		
Yes	309	12.2
No	2218	87.8
Part of healthcare system		
Yes	1564	61.9
No	963	38.1
Number of providers at clinic		
1–5 providers	1155	45.7
6–10 providers	661	26.2
≥ 11 providers	711	28.1
Clinic location		
South	605	23.9
West	841	33.3
Midwest	576	22.8
Northeast	505	20.0

Abbreviations: FQHC, Federally Qualified Health Center.

^a^Includes individual who identified as nonbinary or preferred not to report the gender.

^b^Includes individuals who identified with at least two racial/ethnic groups.

^c^Includes physician assistants, nurse practitioners, advanced practice nurses, clinical nurse specialists.

^d^Includes registered nurses (RNs), licensed practical nurses, licensed vocational nurses, certified nursing assistants, and medical assistants.

Most respondents worked in non-rural clinics (91%), and some primarily worked at a health department (12%). Over one-third of our cohort were part of a larger healthcare system (38%) or worked in a clinic with 1–5 PCHPs (45%). All regions of the US were well-represented, with 24% of participants working in clinics in the South, 33% in the West, 23% in the Midwest, and 20% in the Northeast (Table 1).

### Use of presumptive recommendations

**Fig 1:**
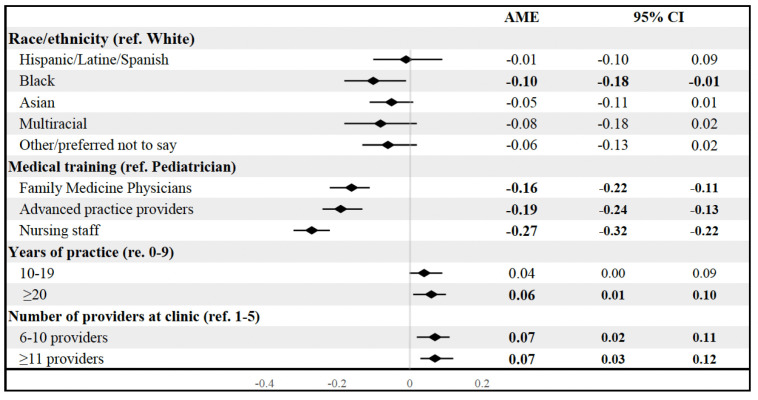
Multivariate associations between primary care professional characteristics and use of the presumptive recommendation. AME, Average marginal effects; CL, confidence limits. Bolded values indicate p < 0.05.

Overall, 58% of our sample reported that the presumptive recommendation was their most often used communication strategy to introduce HPV vaccination with patients. In multivariable analyses, family medicine physicians [AME (95% CI): −0.16 (−0.22, −0.11)], advanced practice providers [AME (95% CI): −0.19 (−0.24, −0.13)], and nursing staff [AME (95% CI): −0.27 (−0.32, −0.22)] were less likely to use the presumptive recommendation compared to pediatricians (Fig 1). Black PCHPs [AME (95% CI): −0.10 (−0.18, −0.01] were less likely to use the presumptive recommendation compared to White PCHPs. PCHPs with more than 20 years of practice were more likely to use the presumptive approach [AME (95% CI): 0.06 (0.01, 0.10)], compared to PCHPs with less than nine years of experience. Finally, PCHPs at clinics with more than 6 providers were more likely to use the presumptive approach than those with one to five providers (Fig 1).

### Strategies used with hesitant parents

Among strategies used with hesitant parents, the majority of PCHPs provided educational materials (80%), offered the vaccine another day (70%), and used prepared talking points (54%). Almost half employed motivational interviewing (48%) and only 27% shared patient stories. In multivariate analyses, Hispanic [AME (95% CI): 0.07 (0.01, 0.09)] and Asian [AME (95% CI): 0.05 (0.01, 0.09)] PCHPs were more likely to provide educational materials than White PCHPs. Multiracial PCHPs were more likely to share patient stories [AME (95% CI): 0.11 (0.01, 0.21)] than White PCHPs. PCHPs with more than 20 years of practice were less likely to offer the vaccine another day [AME (95% CI): −0.06 (−0.11, −0.02)], use prepared talking points [AME (95% CI): −0.07 (−0.12, −0.02)], or use motivational interviewing [AME (95% CI): −0.19 (−0.24, −0.14)] with hesitant parents compared to PCHPs with less than nine years of experience (Table 2).

**Table 2:** Multivariate associations of PCHP characteristics and strategies used with hesitant parents.

	Offer another day		Use prepared talking points		Provided educational materials		Share patient stories		Motivational interviewing	
	AME	95% CL	AME	95% CL	AME	95% CL	AME	95% CL	AME	95% CL
**Gender**										
Man	–	–	Ref.	–	Ref.	–	Ref.	–	–	–
Woman	–	–	0.03	(−0.02, 0.08)	**0.05**	**(0.01, 0.09)**	−0.04	(−0.11, 0.00)	–	–
Another gender	–	–	**−0.17**	**(−0.29, −0.05)**	0.04	(−0.07, 0.15)	−0.11	(−0.22, 0.00)	–	–
**Race/Ethnicity**										
Asian	−0.03	(−0.08, 0.03)	–	–	**0.05**	**(0.01, 0.09)**	0.04	(−0.02, 0.09)	**−0.07**	**(−0.13, −0.02)**
Black	−0.05	(−0.15, 0.04)	–	–	0.05	(−0.03, 0.12)	−0.02	(−0.11, 0.06)	0.07	(−0.02, 0.16)
Hispanic/Latine/Spanish	0.02	(−0.07, 0.12)	–	–	**0.07**	**(0.01, 0.09)**	0.04	(−0.06, 0.13)	−0.04	(−0.14, 0.06)
White	Ref.	–	–	–	Ref.	–	Ref.	–	Ref.	–
Multiracial	**0.09**	**(0.00, 0.18)**	–	–	0.07	(−0.01, 0.14)	**0.11**	**(0.01, 0.21)**	−0.02	(−0.13, 0.08)
Other/preferred not to say	−0.01	(−0.08, 0.07)	–	–	0.01	(−0.07, 0.08)	0.01	(0.01, 0.21)	−0.02	(−0.10, 0.05)
**Medical training**										
Pediatricians	Ref.	–	Ref.	–	Ref.	–	Ref.	–	Ref.	–
Family Medicine Physicians	**−0.06**	**(−0.11, −0.01)**	**−0.11**	**(−0.16, −0.05)**	0.01	(−0.04, 0.06)	0.04	(−0.01, 0.09)	0.04	(−0.02, 0.09)
Advanced practice providers	**−0.06**	**(−0.11, −0.01)**	**−0.07**	**(−0.12, −0.01)**	**0.07**	**(0.03, 0.12)**	0.03	(−0.03, 0.08)	−0.03	(−0.09, 0.03)
Nursing staff	**−0.12**	**(−0.17, −0.07)**	**−0.14**	**(−0.20, −0.08)**	**0.11**	**(0.07, 0.16)**	−0.04	(−0.09, 0.00)	**−0.21**	**(−0.27, −0.16)**
**Patients ages 9–12 seen in a typical week**										
< 10	–	–	–	–	–	–	Ref.	–	Ref.	–
10–24	–	–	–	–	–	–	**0.07**	**(0.03, 0.11)**	0.04	(−0.01, 0.09)
≥ 25	–	–	–	–	–	–	**0.1**	**(0.05, 0.15)**	**−0.03**	**(−0.08, 0.03)**
**Years of practice**										
0–9	Ref.	–	Ref.	–	–	–	–	–	Ref.	–
10–19	−0.03	(−0.07, 0.02)	**−0.06**	**(−0.11, −0.02)**	–	–	–	–	**−0.10**	**(−0.15, −0.05)**
≥ 20	**−0.06**	**(−0.11, −0.02)**	**−0.07**	**(−0.12, −0.02)**	–	–	–	–	**−0.19**	**(−0.24, −0.14)**
**Clinic location**										
South	–	–	–	–	**0.06**	**(0.02, 0.12)**	–	–	−0.01	(−0.06, 0.05)
West	–	–	–	–	0.04	(−0.01, 0.09)	–	–	**0.07**	**(0.01, 0.13)**
Midwest	–	–	–	–	**0.07**	**(0.02, 0.12)**	–	–	0.02	(−0.04, 0.08)
Northeast	–	–	–	–	Ref.	–	–	–	Ref.	–

AME, Average marginal effects; CL, confidence limits.

Bolded values indicate p < 0.05.

Compared to pediatricians, family medicine physicians [AME (95% CI): −0.06 (−0.11, −0.01)], advanced practice providers [AME (95% CI): −0.06 (−0.11, −0.01)], and nursing staff [AME (95% CI): −0.12 (−0.17, −0.07)] were less like to offer the vaccine another day. Family medicine physicians [AME (95% CI): −0.11 (−0.16, −0.05)], advanced practice providers [AME (95% CI): −0.07 (−0.12, −0.01)], and nursing staff [AME (95% CI): −0.14 (−0.20, −0.08)] were also less likely to use prepared talking points with hesitant parents. Nursing staff were less likely (than pediatricians) to employ motivational interviewing techniques [AME (95% CI): −0.21 (−0.27, −0.16)] with hesitant parents. However, advanced practice providers [AME (95% CI): 0.07 (0.03, 0.12)] and nursing staff [AME (95% CI): 0.11 (0.07, 0.16)] were more likely to provide educational materials than pediatricians (Table 2).

### Prior training received & use of the presumptive recommendation

**Fig 2:**
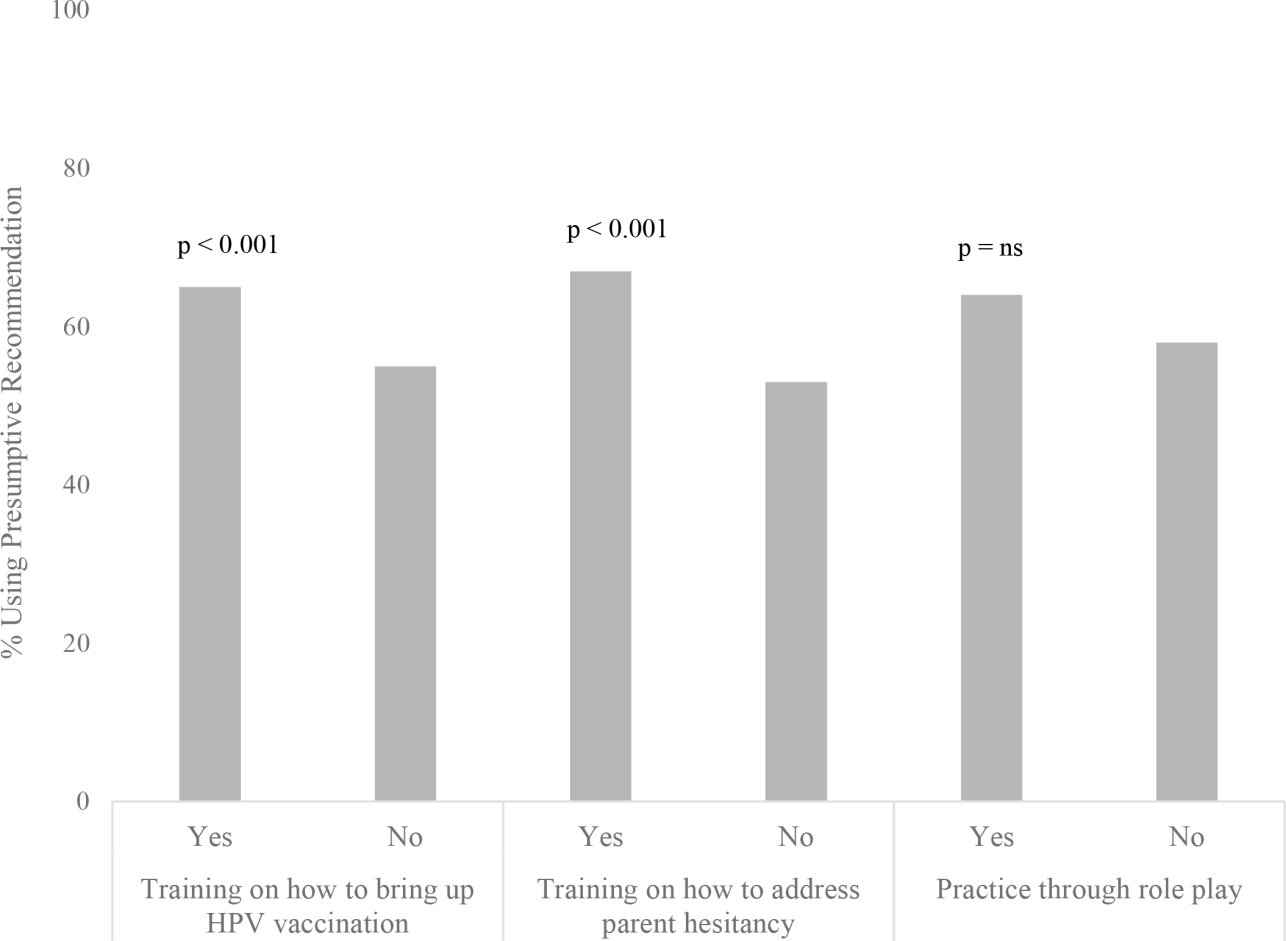
Differences in training by use of presumptive recommendation. ^a^ P-values based on chi-square tests.

PCHPs who had received training in how to bring up HPV vaccination (65% vs. 55%, p < 0.001) and how to address parental hesitancy (67% vs. 53%, p < 0.001) were more likely to use the presumptive approach compared to those who had not received training. PCHPs who had or had not had previous practice through role play did not have significant differences in using the presumptive recommendation (64% vs. 58%, p = 0.09) (Fig 2).

## Discussion

Using data from a national survey of practicing PCHPs involved in HPV vaccination, we examined factors associated with the use of the presumptive recommendation and additional communication strategies used with hesitant parents. We found that use of the presumptive recommendation varied by PCHP race or ethnicity, and medical training. The use of other strategies used with hesitant parents varied primarily based on PCHP’s medical training. Finally, PCHPs who had received training on bringing up HPV vaccination and addressing parental hesitancy were more likely to use presumptive recommendations.

Few studies have found associations between race and HPV vaccine communication strategies recommendations. In a recent systematic review, only one of six studies that stratified the use of high-quality HPV vaccination communication strategies by a PCHPs’ race or ethnicity found a statistically significant association between these variables [15,17–29]. Specifically, one study found that Asian PCHPs were more likely to routinely recommend HPV vaccination to children ages 9–10 compared to White PCHPs [28]. Our study found that Black PCHPs were less likely to use the presumptive recommendation than White PCHPs. The reasons for this difference are unclear. While explanations such as cultural factors or differing approaches to building trust with patients might play a role, there is insufficient empirical evidence to draw conclusions. Future research is needed to contextualize this finding and whether it is potentially related to provider identity, patient population, or clinical context.

The use of the presumptive recommendation and strategies employed with hesitant parents differed by PCHPs’ medical training. Overall, pediatricians were more likely to use strategies that involved direct communication with a hesitant parent, such as motivational interviewing or using prepared talking points. In contrast, non-pediatricians (i.e., advanced practice providers and nursing staff) more often used indirect communication techniques, such as providing educational materials to patients. Differences in use of these strategies may be related to differences in PCHPs’ respective training programs, where HPV communication training and educational opportunities are greater for physicians. For example, one study found that nurse practitioners and physician assistants were more likely to agree with vaccine misperceptions such as ‘vaccinating adolescents against HPV increases the likelihood of unprotected sex’ than family medicine physicians [30]. Increasing HPV vaccine knowledge and increasing training on communication strategies at all clinical roles is vital to increase HPV vaccine uptake. This supports the a ‘whole-office’ approach, which suggests a need for more HPV vaccine knowledge and communication education among all staff [31].

Existing literature shows that multiple communication strategies may be necessary to improve HPV vaccination uptake. Although the presumptive recommendation has been vastly supported by previous studies, new findings support the benefit of using additional communication strategies for encounters that involve hesitant parents. Motivational interviewing, for example, has been an effective component of multi-strategy interventions to improve HPV vaccine uptake when utilized with parents who initially decline vaccination [13].

A few limitations of our study should be noted. The nature of this cross-sectional study only allows us to describe associations as opposed to causal relationships and does not explain why certain PCHPs do or do not employ the presumptive recommendation or other strategies with hesitant parents. Research is needed to better understand PCHP preferences and reasons for employing certain strategies over others. Determining whether these preferred strategies were related to their training, personal preferences, perceived patient population needs, and/or clinical time restraints is necessary for implementing a team-based approach to HPV vaccine delivery. Moreover, the participants who chose to contribute to this online survey may have characteristics that are not representative of the larger population of US PCHPs. Although our results are strengthened by the variability of geographic setting, training background, and years of experience of participants, we are unable to compare their characteristics to those who chose not to participate. The voluntary nature of study participation introduces selection bias, and limits the generalizability of our study results. Data on use of strategies is subject to self-reporting bias, where participants may report based on inaccurate self-assessments or social desirability. While data for this analysis was from a nationally-based survey, the participant sample may not be nationally representative. Most participants were women, and from urban or suburban areas. The majority of our sample self-identified as White. However, our sample had a diversity of training backgrounds, represented a wide geographic distribution, and included PCHPs with differences in years of clinical experience. Thus, our findings offer insights into the PCHP characteristics related to participants’ use of the presumptive recommendation and other strategies to support HPV vaccine uptake.

## Conclusion

The presumptive recommendation, prepared talking points, educational materials, offering the vaccine another day, motivational interviewing, and story-sharing are communication tools used by many PCHPs to promote HPV vaccination. However, in alignment with prior research, use of these strategies may vary depending on PCHPs’ characteristics, particularly their medical training. Our findings highlight differences in use of vaccine communication techniques among PCHPs with varying individual-level characteristics. Interventions are needed to ensure that PCHPs of all medical training backgrounds are trained and empowered to utilize evidence-based communication strategies to improve HPV vaccine uptake.

## Supporting information

S1 File Please see average marginal effects and 95% confidence intervals for associated covariates in the supplemental file. (DOCX)
