# Supplementary material for: Use of presumptive recommendations and other strategies to encourage HPV vaccine uptake: Results from a national survey of primary care health professionals
Source: PLoS One. 2025 Aug 4;20(8):e0327872. doi: 10.1371/journal.pone.0327872 (PMC12321062; doi:10.1371/journal.pone.0327872)
Supplement: S1 File — (DOCX) [file pone.0327872.s001.docx]

**Supplemental Files**

| **Supplemental Table 1.** Bivariate associations between primary care professional characteristics and use of the presumptive approach | | | | | |
| --- | --- | --- | --- | --- | --- |
|  | **n/N** | **%** | **AME** | **95% CL** | |
| **Gender** |  |  |  |  |  |
| Man | 368/637 | 58 | Ref. |  |  |
| Woman | 1071/1810 | 59 | 0.01 | -0.03 | 0.06 |
| Another gender^a^ | 39/80 | 49 | 0.09 | -0.21 | 0.03 |
| **Race/Ethnicity** |  |  |  |  |  |
| Asian | 209/356 | 59 | -0.01 | -0.07 | -0.04 |
| Black | 61/123 | 50 | **-0.10** | **-0.19** | **-0.01** |
| Hispanic/Latine/Spanish | 58/100 | 58 | -0.02 | -0.12 | 0.08 |
| White | 996/1664 | 60 | Ref. |  |  |
| Multiracial | 49/94 | 52 | -0.08 | -0.18 | 0.03 |
| Other/preferred not to say | 105/190 | 55 | -0.05 | -0.12 | 0.03 |
| **Medical training** |  |  |  |  |  |
| Physicians |  |  |  |  |  |
| Pediatrician | 496/666 | 74 | Ref. |  |  |
| Family medicine | 318/557 | 57 | **-0.17** | **-0.23** | **-0.12** |
| Advanced practice providers | 326/603 | 54 | **-0.20** | **-0.26** | **-0.15** |
| Nursing staff | 338/701 | 48 | **-0.26** | **-0.31** | **-0.21** |
| **Patients ages 9-12 seen in a typical week** |  |  |  |  |  |
| <10 | 408/730 | 56 | Ref. |  |  |
| 10 - 19 | 595/1000 | 60 | 0.04 | -0.01 | 0.08 |
| ≥25 | 475/797 | 60 | 0.04 | -0.01 | 0.09 |
| **Years of practice** |  |  |  |  |  |
| 0-9 | 523/950 | 55 | Ref. |  |  |
| 10-19 | 438/740 | 59 | 0.04 | -0.01 | 0.09 |
| ≥20 | 517/837 | 62 | **0.07** | **0.02** | **0.11** |
| **Number of providers at clinic** |  |  |  |  |  |
| 1-5 providers | 625/1155 | 54 | Ref. |  |  |
| 6-10 providers | 407/661 | 62 | **0.07** | **0.03** | **0.12** |
| ≥11 providers | 446/711 | 63 | **0.09** | **0.04** | **0.13** |
| **Clinic location** |  |  |  |  |  |
| South | 387/605 | 64 | -0.16 | -0.38 | 0.06 |
| West | 458/841 | 54 | 0.23 | -0.01 | 0.52 |
| Midwest | 338/576 | 59 | 0.01 | -0.23 | 0.25 |
| Northeast | 295/505 | 58 | Ref. |  |  |
| AME, Average marginal effects; CL, confidence limits | | | | | |
| Bolded values indicate p<0.05 | | | | | |
